# Antenatally detected cystic biliary atresia: differential diagnoses of a double bubble

**DOI:** 10.1186/2193-1801-3-368

**Published:** 2014-07-19

**Authors:** Victoria A Adewole, Naomi J Wright, Ruth Hallows, Mark Davenport

**Affiliations:** Department of Paediatric Surgery, Royal Alexandra Children’s Hospital, Eastern Road, Brighton, BN2 5BE England; Department of Paediatric Surgery, King’s College Hospital, Denmark Hill, London, SE5 9RS USA

**Keywords:** Cystic biliary atresia, Antenatal ultrasound, “double bubble”

## Abstract

The ‘double bubble’ sign on antenatal ultrasound is often associated with duodenal atresia although there are numerous causes. We present a case of cystic biliary atresia presenting with a “double bubble” at 36-weeks gestation. Postnatal ultrasound and MRCP confirmed a cystic lesion at the porta hepatis, mandating early laparotomy and a successful Kasai portoenterostomy.

Although diagnosis of such lesions may be imprecise antenatally, awareness and detection does allow early postnatal investigation and management, which is vital to optimise outcome.

This case highlights the need to be mindful of other important anomalies that can give this appearance and that may require early intervention.

## Introduction

Fetal anomaly scanning is key to the diagnosis of various congenital conditions. It is highly sensitive for the detection of abdominal wall defects (e.g. gastroschisis) and duodenal/ proximal intestinal atresias, but is less so for a number of other abdominal conditions. In addition, ultrasonography (US) can be used to identify extra abdominal pathology such as congenital diaphragmatic herniae and hydrocephalus.

The ‘double bubble’ sign seen and originally described on plain radiography, but now also appreciable on US, is a result of fluid-filled structures seen in the either hypochondrium or epigastrium. Historically this sign has been strongly associated with duodenal atresia or other causes of duodenal obstruction. Nonetheless, to consider this sign as pathognomonic for duodenal atresia is dangerous and it is important to be mindful of other important anomalies that can give this appearance and that may also require urgent intervention.

The parents of the child involved have kindly given us their written consent to share the case with our colleagues. Our aim is to remind clinicians of an important pathology.

## Case report

**Figure 1:**
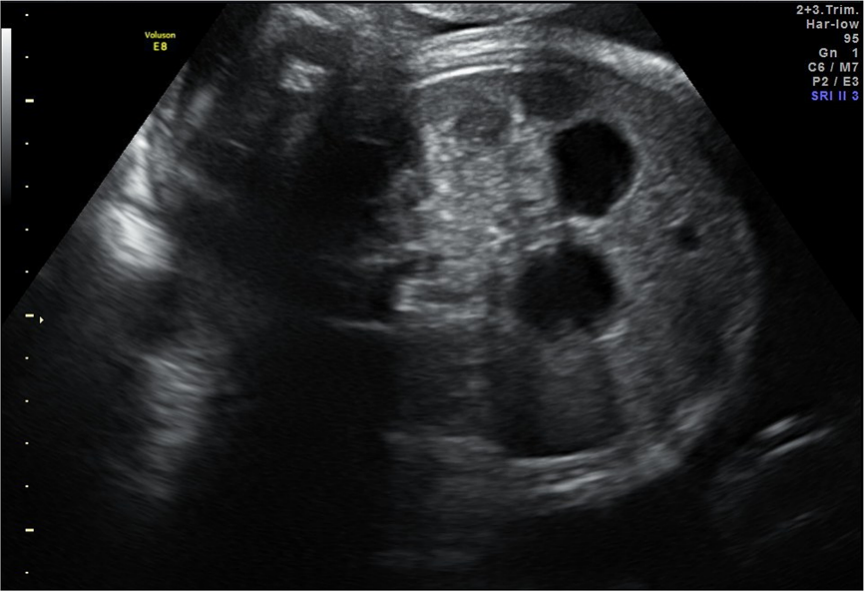
**Antenatal ultrasound at 36 weeks gestation.** Two fluid filled structures in the fetal abdomen: a ‘double bubble’ sign.

**Figure 2:**
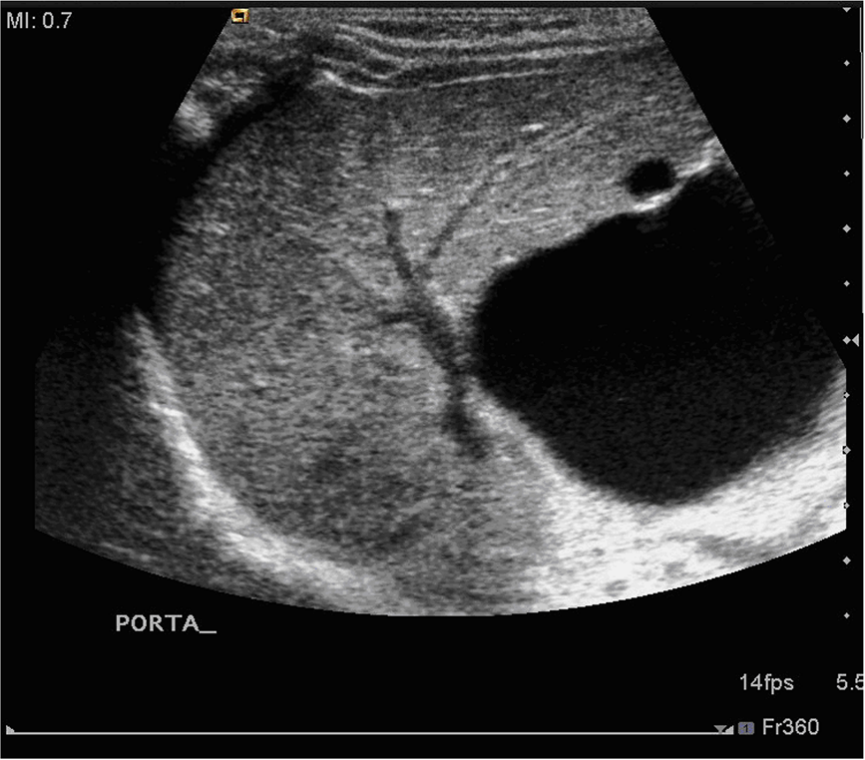
**Abdominal ultrasound of the right upper quadrant.** A large anechoic cystic lesion is seen outside the liver, in the region of the porta hepatis, measuring 38 mm. Another smaller extrahepatic cyst is seen. There is no intrahepatic duct dilatation.

A male infant born at term was referred to our tertiary paediatric surgery centre at 2 days old with a history of bile-stained nasogastric aspirates. Although his 20-week fetal anomaly scan had been normal, a maternal US scan at 36 weeks for high fundal height and suspected polyhydramnios showed the appearance of a “double bubble” (Figure 1), but without polyhydramnios.The child was well on arrival, aspirates were clear and nothing abnormal could be palpated in his abdomen. An upper gastrointestinal contrast study showed free drainage into a normally-rotated, non-dilated duodenum and jejunum. An abdominal US identified a cystic structure measuring 38 mm in diameter in the region of the porta hepatis of uncertain aetiology (Figure 2).

Enteral feeding was started and tolerated well. However, within the week, nursing staff noticed that the stools were pale.

**Figure 3:**
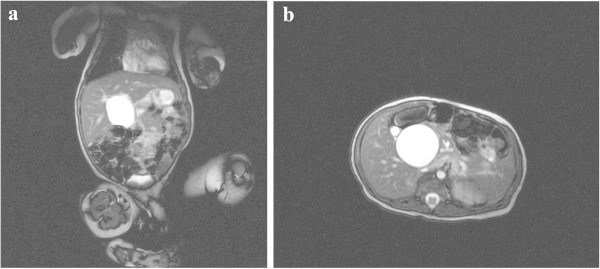
**Coronal plane (a) and Transverse plane (b) T2-W Trufi Magnetic Resonance Cholangiopancreatography at 8 days old.** Two cystic lesions are demonstrated in the extrahepatic biliary tree, largest measuring 32.8 mm x 40 mm. There is no intrahepatic biliary dilatation and a lack of continuity of the extrahepatic biliary tree with the duodenum, suggesting a diagnosis of CBA rather than a choledochal cyst.

**Figure 4:**
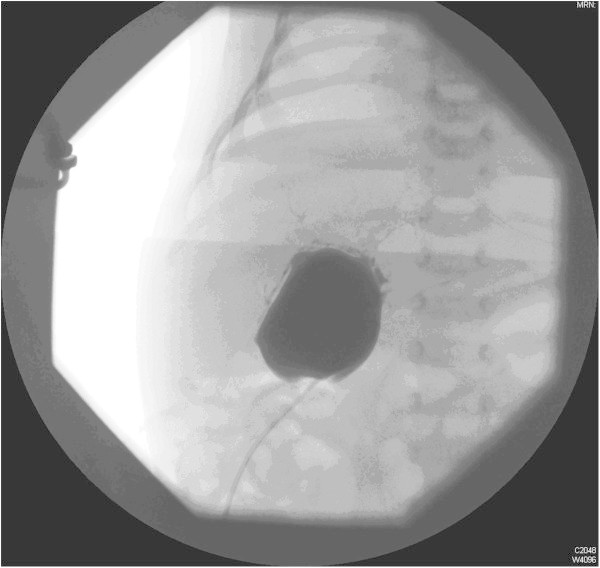
**Cholangiogram showing large cystic dilatation and abnormal etiolated, hypoplastic intrahepatic biliary ducts consistent with cystic biliary atresia.**

Liver biochemistry at day 9 showed total bilirubin of 113 μmol/L with a conjugated bilirubin of 83.9 μmol/L (74% of total); alkaline phosphatase (ALP) 280 iu/L (normal <449 iu/L) and alanine transferase 71 iu/L (reference range <41 iu/L).Magnetic Resonance Cholangiopancreatography (MRCP) was requested to evaluate the biliary system (Figure 3a and b) and showed a 32.8 mm × 40 mm cyst in the region of the porta hepatis. He was then transferred to a specialist paediatric liver centre where a laparotomy was carried out on day 20. This showed a mucus-containing cyst which on intra-operative cholangiography (Figure 4) had a tenuous connection to a non-dilated irregular biliary tree. The transected porta hepatis did not show any visible bile duct consistent with Type III BA (Japanese Association of Pediatric Surgeons (JAPS) classification). A Kasai-type portoenterostomy was performed following which bile flow was restored and his jaundice cleared. He is now 4 months old, thriving and jaundice free.

## Discussion

Biliary atresia (BA) is a disease of unknown aetiology with an incidence in the UK of about 1 in 17,000 live births (Livesey et al. 2009). CBA, characterized by dilatation within an otherwise obliterated extrahepatic biliary tree, occurs in about 5% of large series and is detectable on maternal US (Davenport and Hadzic 2003; Caponcelli et al. 2008). Conjugated jaundice and pale stools in the neonatal period are invariable. The JAPS classification is based on the level of the most proximal obstruction: thus type I is at the level of the common bile duct; type II, at the level of the common hepatic duct; and type III (this case) at the level of the porta hepatis.

Identification of a “double bubble” on maternal US mandates a search for possible causes post-natally (Table 1). In this case, an upper GI contrast was performed to assess for the commonest causes - duodenal atresia, stenosis or obstruction due to malrotation or annular pancreas. The latter may be suggested by identifying a hyperechogenic band around the duodenum in addition to the double bubble (Dankovcik et al. 2008). Gastrointestinal duplication cysts can be identified on ultrasonography as an anechoic cystic lesion that is separate from normal hollow structures, typically with a double wall.

**Table 1:** **Differential diagnoses to consider when the double bubble sign is seen on antenatal ultrasound**

Site of pathology	Differential diagnoses
Luminal obstruction	Duodenal atresia
	Duodenal stenosis
	Jejunal atresia
Extra-luminal obstruction	Intestinal malrotation
	Annular pancreas
	Diaphragmatic hernia
Hepatobiliary	Cystic choledochal malformation
	Developmental hepatic cyst
	Cystic biliary atresia
Other GI	Duplication cyst
Non-gastrointestinal	Omental cyst, ovarian cyst, renal cyst
Non-pathological	Transient bubble associated with slow peristalsis

In this instance, the double bubble on antenatal ultrasonography, was in fact created by the extrahepatic cyst and the fetal stomach.

Antenatal diagnosis of hepatobiliary disease is notoriously difficult and rarely correct (Davenport and Hadzic 2003). However, cystic biliary malformations such as CBA or cystic choledochal malformation should remain an important differential particularly if, postnatally, there is clinical evidence of cholestasis and absence of bile in the GI tract. Post-natal differentiation between these two pathologies may be particularly difficult if the latter is obstructed. The cystic element tends to be smaller in CBA and sometimes the intrahepatic bile ducts dilate in an obstructed choledochal malformation. This latter feature never happens in BA. Intuitively it might be thought that non-visualization of the gallbladder on a neonatal US scan might suggest BA. However, this is seldom the case and Farrant et al. showed that a gallbladder is visible in up to 94% of BA cases in their series. Although an abnormal appearance was very common (Farrant et al. 2001).

In this child’s case biliary malformations were not considered in the antenatal period. This is not uncommon. In the Kings College Hospital experience of neonates and infants with CBA and abnormal antenatal scans, none of the children had the correct diagnosis amongst the prenatal differentials (Davenport and Hadzic 2003). However, an accurate diagnosis was possible on the postnatal imaging, but not always considered. This highlights the lack of awareness of this disease presentation even though it was first reported in the literature in 1986. Thus it is important for those looking at both antenatal and postnatal imaging to be aware of this pathology as early surgery is key to optimising outcome. While a choledochal cyst was considered briefly by our local radiologist after the post natal US, the liver function tests being considered to be only ‘mildly’ abnormal, confused the diagnosis, with the total bilirubin being below the treatment line for a neonate within the first week of life. A split bilirubin was not requested until after this, on day 9, and so the obstructive picture was masked until then.

US does have a key role in the post-natal investigation of a persistently jaundiced infant, either to confirm the presence of choledochal malformation, inspissated bile syndrome or spontaneous biliary perforation; or in the actual diagnosis of BA. In non-cystic BA, the “triangular cord” sign has been advocated as a reliable diagnostic sign (Humphrey and Stringer 2007). It can be defined as an echogenic appearance anterior to the wall of the right portal vein of >4 mm on longitudinal scan and corresponds to the obliterated proximal remnant in the porta hepatis. In a large series from Korea, Lee et al. showed it to have a sensitivity of 80%, and specificity of 98% in non-cystic BA (Lee et al. 2003). Similarly, Zhou et al. showed in a small study of 23 patients that it may also be useful when differentiating between CBA and choledochal cysts. In this series the triangular cord sign had a sensitivity of over 90% and 100% specific in identifying CBA.

**Figure 5:**
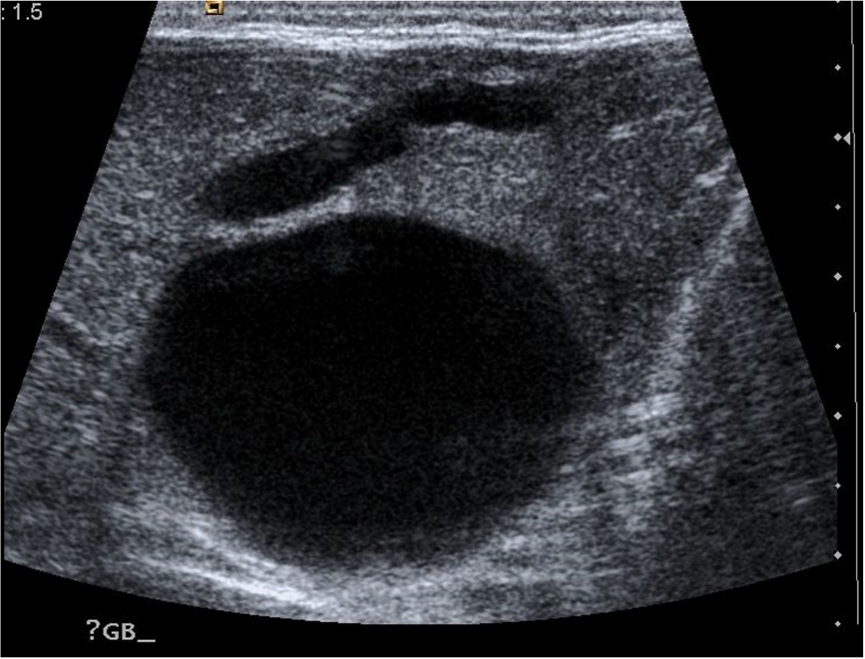
**Post natal abdominal US scan.** Shows the cystic lesion and a gallbladder that is seen to be convoluted and abnormal in position and morphology.

The group also suggested, as much of the literature in the case of noncystic BA, that other features such as dilatation of the intrahepatic bile ducts and hepatic artery; the sizes and morphologic characteristics of the gallbladder and liver may also be helpful identifying these cases (Zhou et al. 2012). When taking these into account they were able to correctly differentiate all the CBA cases from those with choledochal cysts. Looking for these on post natal abdominal US may have aided the diagnosis. Retrospectively the gallbladder was noted to look abnormal on his post natal US scan (Figure 5).

A management algorithm has been previously suggested in which confirming the cyst along with a non-dilated intrahepatic biliary tree and deranged liver biochemistry lead to urgent laparotomy and reconstructive surgery (Davenport and Hadzic 2003). It is important to note that although the total bilirubin was initially considered below the treatment line for a week old 1 infant, the conjugated fraction was actually considerably elevated. Levels of > 20% of the total are pathological and indicate the need for further investigation.

Although the influence of age on outcome following surgery in BA is controversial, it is incontestable in the CBA variant. Caponcelli et al. showed a clear relationship between age at portoenterostomy and clearance of jaundice in a series of 29 infants with CBA. All those operated on at <40 days cleared their jaundice compared to none at >70 days (Caponcelli et al. 2008).

## Conclusion

With the advances in antenatal scanning, there is the opportunity to offer early intervention for potentially serious hepatobiliary disease in the neonate. Currently antenatal diagnosis of cystic biliary malformations is imprecise, which may be permissible as antenatal intervention is not warranted. However, antenatal detection does mandate early postnatal investigation. This can allow for an early diagnosis and treatment, but only if clinicians are mindful of the possible diagnoses. The first step in this is recognising that cystic biliary malformations, and crucially CBA, may be a cause of the “double bubble” sign on antenatal US.

## Consent

Written informed consent was obtained from the patient’s guardian/parent/next of kin for the publication of this report and any accompanying images.
